# Analysis of human sperm DNA fragmentation index (DFI) related factors: a report of 1010 subfertile men in China

**DOI:** 10.1186/s12958-018-0345-y

**Published:** 2018-03-14

**Authors:** Jin-Chun Lu, Jun Jing, Li Chen, Yi-Feng Ge, Rui-Xiang Feng, Yuan-Jiao Liang, Bing Yao

**Affiliations:** 1 The Reproductive Medical Centre, Nanjing Jinling Hospital, Nanjing University School of Medicine, 305 Zhongshan East Road, Nanjing, 210002 Jiangsu China; 2 Department of Laboratory Science, Nanjing Hospital, Jiangsu Corps, The Armed Police Force, PLA, Nanjing, 210028 Jiangsu China

**Keywords:** Sperm DNA fragmentation index, Male infertility, Obesity-associated marker, Lipids, Reproductive hormone

## Abstract

**Background:**

Many factors may lead to sperm DNA damage. However, it is little known that the correlations of sperm DNA damage with obesity-associated markers, and reproductive hormones and lipids levels in serum and seminal plasma.

**Methods:**

In our prospective study, a total of 1 010 subfertile men, aged from 18 to 50 years old, were enrolled from August 2012 through June 2015. Their obesity-associated markers, semen parameters, sperm acrosomal enzyme activity, seminal plasma biochemical markers, and reproductive hormones and lipids levels in serum and seminal plasma were detected. Sperm DNA fragmentation index (DFI) was determined by sperm chromatin structure assay. The correlations between DFI and each of the above-mentioned variables were analyzed.

**Results:**

Spearman correlation analysis showed that sperm DFI was positively related to age and abstinence time (*P*<0.001). Sperm DFI was also positively related to semen volume and percent of abnormal sperm head (*P*<0.001), while negatively related to sperm concentration, progressive motility (PR), sperm motility, total normal-progressively motile sperm count (TNPMS), percent of normal sperm morphology (NSM), percent of intact acrosome and acrosomal enzyme activity (*P*<0.001). Sperm DFI was positively related to seminal plasma zinc level (*P*<0.001) but unrelated to seminal plasma total α-glucotase, γ-glutamyl transpeptidase (GGT) and fructose levels. There was no any correlation between sperm DFI and obesity-associated markers such as body mass index (BMI), waist-to-hip ratio (WHR), waist circumference (WC) and waist-to-height ratio (WHtR), and serum lipids levels, but there was positive correlation between sperm DFI and seminal plasma triglyceride (TG) and total cholesterol (TC) levels (*P*<0.001). Sperm DFI was positively related to serum luteinizing hormone (LH) and follicle stimulating hormone (FSH) levels and seminal plasma FSH and estradiol (E2) levels (*P*<0.001), but unrelated to serum and seminal plasma testosterone (T) levels. The multivariate regression analysis for the variables which were significantly correlated with sperm DFI in Spearman correlation analysis showed that age, semen volume, sperm concentration, progressive motility, TNPMS and intact acrosome were independently correlated with sperm DFI.

**Conclusions:**

There are many potential factors associated with sperm DFI, including age, abstinence time, spermatogenesis and maturation, seminal plasma lipids and reproductive hormones levels. However, the potential effects of seminal plasma lipids and reproductive hormones on sperm DNA damage need still to be demonstrated by the studies with scientific design and a large size of samples.

**Electronic supplementary material:**

The online version of this article (10.1186/s12958-018-0345-y) contains supplementary material, which is available to authorized users.

## Background

Through searches of “sperm” and “DNA fragmentation index” in PubMed from 2002 to August 2015, we retrieved over 200 literatures associated with sperm DNA fragmentation index (DFI). Comprehensive analyses of these literatures indicated that since the detection of sperm DNA damage was performed in 2002, it had been applied in some clinical andrology laboratories. The detection of sperm DNA damage, as an important supplement to semen routine examination strategies, may predict the outcomes of natural conception and *in vitro* fertilization, monitor the damage of sperm DNA induced by environmental pollutants and medical interventions, and evaluate the sperm DNA damage related to male reproductive system diseases and their treatments [1]. The factors related to sperm DNA damage included age, environmental pollutants such as organophosphorus and organochloride pesticides, plasticizer, heavy metals such as lead, carcinogens such as polycyclic aromatic hydrocarbons (c-PAHs) and zearalenone (ZEA), male reproductive system diseases or systemic diseases such as varicocele, infection, tumor, spermatogenesis and maturation dysfunction, spinal cord injury and endocrine disorders, seasons and temperature, lifestyle, abstinence time, semen refrigeration, semen handling *in vitro*, and certain medications [2]. However, controversial results remain.

Obesity and subfertility have become major global public health concerns. Obesity was reportedly associated with lower fertility. Our previously published data showed that, although obesity-associated markers such as body mass index (BMI), waist circumference (WC), waist-to-hip ratio (WHR) and waist-to-height ratio (WHtR) could not predict semen quality [3], the metabolism abnormality of lipids in male reproductive system may affect male fertility [4]. It raises the question of whether sperm DNA damage, as an important factor influencing sperm quality, be associated with obesity and metabolism abnormality of lipids. No data currently available address this question [5]. In this study, we comprehensively analyzed the correlations between sperm DFI and age, abstinence time, obesity-associated markers such as BMI, WC, WHR and WHtR, semen parameters such as semen volume, sperm concentration, total sperm count (TSC), sperm motility, progressive motility (PR), percent of normal sperm morphology (NSM), percent of abnormal sperm head, percent of intact acrosome, acrosomal enzyme activity and total normal-progressively motile sperm count (TNPMS), serum lipids such as total cholesterol (TC), triglyceride (TG), low density lipoprotein cholesterol (LDL-C) and high density lipoprotein cholesterol (HDL-C) levels, serum reproductive hormones such as follicle stimulating hormone (FSH), luteinizing hormone (LH), estradiol (E2), total testosterone (TT) and sex hormone binding globulin (SHBG) levels, seminal plasma biochemical markers such as total α-glucotase, γ-glutamyl transpeptidase (GGT), zinc and fructose, seminal plasma lipids such as TG, TC, LDL-C and HDL-C, and seminal plasma reproductive hormones such as FSH, E2 and testosterone in 1 010 subfertile Chinese men (The raw data for these variables were shown in Additional file 1).

## Methods

### Study population

Subfertile men, aged from 18 to 50 years and whose partners had not conceived within 12 months after stopping use of contraception, who attended infertility outpatient clinic at Nanjing Jinling Hospital between August 2012 and June 2015 were included in this prospective study. This study was approved by the Human Subject Committees of Nanjing Jinling Hospital (Approved number: 2012NZKY-012), and informed consent was obtained from all participants. All participants were asked to complete a questionnaire to provide information on occupation, medical and reproductive history and lifestyle factors including intake of alcohol and smoking history. Then, all participants underwent physical examination, and obesity-associated markers were measured, semen samples were collected, and fasting venous blood were drawn during 8:00 am and 10:00 am. Stringent exclusion criteria were employed to exclude regular alcohol drinkers, heavy smokers, and men with chronic diseases, urogenital infections, varicocele, azoospermia and other diseases which might lead to dysspermia, and incomplete data. One thousand and ten (1 010) men were eligible for the inclusion criteria and enrolled in this study.

### Measurement of obesity-associated markers

Height and weight were measured with the participants standing without shoes and heavy outer garments. WC was measured at the level midway between the lower rib margin and the iliac crest with participants in standing position without heavy outer garments and with empty pockets, breathing out gently. Hip circumference was recorded as the maximum circumference over the buttocks. BMI was calculated as weight divided by height squared (kg/m^2^). WHR was calculated as the ratio of WC over the hip circumference. WHtR was calculated as the ratio of WC over height.

With regard to the current Chinese men criteria [6], a BMI under 18.5 kg/m^2^ was considered underweight; BMI between 18.5 and 23.99 kg/m^2^ as normal weight; BMI between 24 and 27.99 kg/m^2^ as overweight; and BMI ≥ 28 kg/m^2^ as obesity. Generalized obesity and abdominal obesity were defined using WHO Asia Pacific guidelines with WC cutoff as ≥ 90 cm [7], WHR cutoff as ≥ 0.9 [8], and WHtR cutoff as ≥0.5 [9].

### Analysis of semen parameters

Semen specimens were collected by masturbation after a period of 2–7 days of sexual abstinence and were kept to liquefy at 37 °C for 30 min. After liquefaction, semen volume was measured by weighing the sample, sperm concentration, total motility and PR were analyzed using a computer-aided sperm analysis (CASA) system (CFT-9201; Jiangsu Rich Life Science Instrument Co., Ltd., Nanjing, China) [10], and sperm morphology was evaluated using Diff-Quik staining, and NSM, percent of abnormal sperm head, percent of intact acrosome and TNPMS (semen volume × sperm concentration × PR × NSM) were calculated. Here TNPMS represents the spermatozoa with motility and normal morphology, and it is a determinative factor for male fertility. For each specimen, at least 200 spermatozoa were counted and analyzed in each replicate. If the difference between the two replicates was acceptable (within 95% confidence interval), the average results of sperm concentration, total motility, PR and NSM were reported. If the difference was too high, two new aliquots from the semen sample were repeatedly assessed [11]. Based on a colorimetric method, the determination of acrosomal enzyme activity was performed strictly according to the manufacturer’s instruction. The kit was purchased from Nanjing Xindi Biological Pharmaceutical Engineering Co., Ltd. (Nanjing, China).

### Detection of sperm DFI

Sperm DFI was assessed by the sperm chromatin structure assay (SCSA). The SCSA kit was purchased from CellPro Biotech Co., Ltd. (Ningbo, China). The determination of sperm DFI was performed strictly according to the manufacturer’s instruction. In brief, semen samples were treated for 30 s with 400 μl of a solution of 0.1 % Triton X-100, 0.15 mol/L NaCl, and 0.08 mol/L HCl, pH 1.2. After 30 s, 1.2 ml of staining buffer (6 μg/ml acridine orange [AO], 37 mmol/L citric acid, 126 mmol/L Na_2_HPO_4_, 1 mmol/L disodium EDTA, 0.15 mol/L NaCl, pH 6.0) was admixed to the test tube. The sample was placed into the FACS Calibur flow cytometer (Becton Dickinson, San Jose, CA) with the sample flowing to establish optimal sheath/sample flow, and then at exactly 3 min AO staining measurements were taken. A minimum of 5 000 cells from two aliquots of each sample were acquired and analyzed by FACS scan interfaced with a data analysis software (DFIView 2010 Alpha11.15, CellPro Biotech, Ningbo, China). After completion of the sample analysis, the cytogram (red *vs* green fluorescence) and histogram (total cells *vs* DFI) plots as well as DFI readings were generated. A mean of the two sperm DFI values was reported. The variability of the replicate DFI measures was less than 5%.

### Determination of seminal plasma biochemical markers

After liquefaction, routine analysis of each semen sample was performed, and the remaining semen samples were centrifuged at 12 000 g for 5 min. The upper layer seminal plasma was collected for the determination of biochemical markers. Commercially available kits for the determinations of seminal plasma GGT, total α-glucotase, zinc and fructose were purchased from Nanjing Xindi Biological Pharmaceutical Engineering Co., Ltd. (Nanjing, China). The determinations were carried out using the Olympus AU400 automatic biochemistry analyzer (Olympus Optical Co. Ltd., Japan), and performed strictly according to the manufacturer’s instruction [12–15].

### Determinations of serum lipids and reproductive hormones

Fasting venous blood samples were centrifuged at 3 000 g for 5 min to isolate serum for the detections of lipids and reproductive hormones levels. Commercially available kits for the determinations of TG, TC, LDL and HDL were purchased from Shanghai Zhicheng Biotechnology Co., Ltd., China. Calibration and quality control products were purchased from Randox Laboratories Ltd., Northern Ireland, United Kingdom. The determinations of serum lipids were carried out using the Olympus AU400 automatic biochemistry analyzer (Olympus Optical Co. Ltd., Japan). The sample with higher lipid level exceeding the linear range of the kit was diluted with normal saline and the diluted volume was calculated.

Commercially available kits for the determinations of FSH, LH, TT, E2 and SHBG were purchased from Beckman Coulter, Inc., USA. Serum TT, LH, FSH, E2 and SHBG levels were determined by chemiluminescence assay using an automated Unicel Dxi 800 Access Immunoassay System (Beckman Coulter, Inc., USA). The assay sensitivities were 0.35 nmol/L for TT, 0.2 IU/L for LH, 0.2 IU/L for FSH, 73 pmol/L for E2 and 0.33 nmol/L for SHBG. The intra-assay coefficients of variation (CV) for LH, FSH, TT, E2 and SHBG were all less than 5%, and the inter-assay CVs were all less than 8%.

### Determinations of seminal plasma lipids and reproductive hormones

After liquefaction, the routine analysis of each semen sample was performed, and the remaining was centrifuged at 12 000 g for 5 min. The upper layer seminal plasma was collected for the determinations of lipids and reproductive hormones [4]. Commercially available kits for the determinations of TG, TC, LDL and HDL were purchased from Shanghai Zhicheng Biotechnology Co., Ltd., China. Calibration and quality control products were purchased from Randox Laboratories Ltd., Northern Ireland, United Kingdom. Determination of lipids in seminal plasma was carried out using the Olympus AU400 automatic biochemistry analyzer (Olympus Optical Co. Ltd., Japan). For lower LH level in seminal plasma, we didn’t obtain its information. So, we only determined seminal plasma FSH, TT and E2 levels. Commercially available kits for the determinations of FSH, TT and E2 were purchased from Beckman Coulter, Inc., USA. Seminal plasma FSH, TT and E2 levels were determined by chemiluminescence assay using an automated Unicel Dxi 800 Access Immunoassay System (Beckman Coulter, Inc., USA). The sample with higher or lower level exceeding the linear range of the kit was diluted with normal saline or added sample size.

### Statistical analysis

All data analyses were conducted using SPSS 11.0 software (SPSS Inc., Chicago, IL, USA). First, nonparametric tests (one-sample Kolmogorov–Smirnov test) were used to determine whether analyzed parameters were normally distributed. If the parameter was consistent with normal distribution, correlations between sperm DFI and age, obesity-associated markers, semen parameters, seminal plasma biochemical markers, and serum and seminal plasma lipids and reproductive hormones levels were examined by Pearson test. If the parameter was consistent with skewed distribution, correlations were examined by Spearman’s rho test. The variables which were significantly correlated with sperm DFI in Spearman’s rho test were further assessed by the multivariate logistic regression analysis. The differences among different groups and between two groups with different number of samples were assessed by one-way ANOVA test and independent-samples *t* test, respectively. *P*-value < 0.05 was considered statistically significant.

## Results

**Fig. 1:**
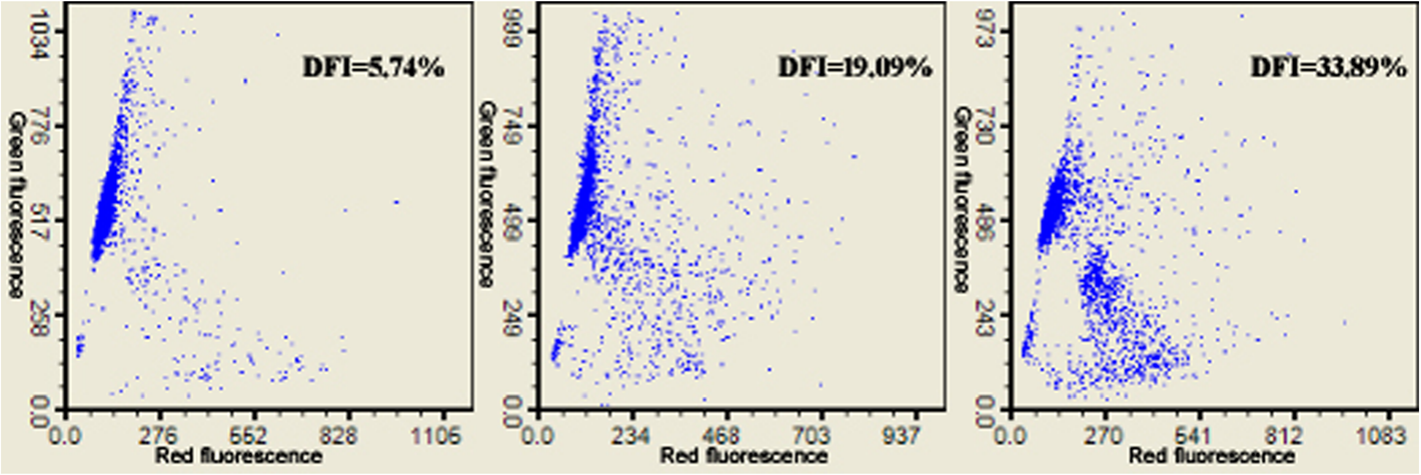
Sperm DFI detected by flow cytometry. Sperm DFI was assessed by the sperm chromatin structure assay (SCSA). In brief, semen samples were treated for 30 s with 400 μl of a solution of 0.1 % Triton X-100, 0.15 mol/L NaCl, and 0.08 mol/L HCl, pH 1.2. After 30 s, 1.2 ml of staining buffer (6 μg/ml acridine orange [AO], 37 mmol/L citric acid, 126 mmol/L Na_2_HPO_4_, 1 mmol/L disodium EDTA, 0.15 mol/L NaCl, pH 6.0) was admixed to the test tube. The sample was placed into the FACS Calibur flow cytometer with the sample flowing to establish optimal sheath/sample flow, and then at exactly 3 min AO staining measurements were taken. A minimum of 5 000 cells from two aliquots of each sample were acquired and analyzed by FACS scan interfaced with a data analysis software. After completion of the sample analysis, the cytogram (red *vs* green fluorescence) and DFI readings were generated.

The results of obesity-associated markers and sperm DFI were obtained from all of 1 010 subfertile men. The numbers of men with intact and effective semen parameters (32 men with 100% of spermatia or 100% of teratospermia were excluded), seminal plasma biochemical markers, serum lipids, serum reproductive hormones, seminal plasma lipids and seminal plasma reproductive hormones were 978, 959, 974, 954, 887 and 396, respectively. Table 1 showed the mean, standard deviation and range of all these variables. The representative figure of sperm DFI was shown in Fig. 1.

**Table 1:** The mean, standard deviation and range of the investigated parameters in 1 010 subfertile men

Variables	Number	Mean (SD)	Range
Age (years)	1010	28.89 (4.60)	18-50
DFI (%)	1010	18.61 (12.46)	1.61-85.78
BMI (kg/m^2^)	1010	23.98 (3.08)	17.36-41.03
WC (cm)	1010	82.14 (9.27)	60-131
WHR	1010	0.86 (0.06)	0.70-1.13
WHtR	1010	0.47 (0.05)	0.34-0.74
Abstinence time (day)	1010	4.28 (1.72)	1-20
Semen volume (ml)	978	3.79 (1.42)	1.1-12
Sperm concentration (10^6^/ml)	978	60.78 (51.97)	0.67-340.44
Total sperm count (10^6^/ejaculate)	978	220.95 (199.10)	1.35-1364.86
Progressive motility (%)	978	32.29 (12.93)	0.91-81.60
Sperm motility (%)	978	45.37 (19.22)	1.51-98.89
Normal sperm morphology (%)	978	4.52 (2.01)	0.41-17.56
TNPMS (10^6^/ejaculate)	978	3.81 (5.00)	0.01-45.20
Abnormal head (%)	978	67.84 (7.86)	11.10-95.15
Intact acrosome (%)	978	57.00 (10.61)	8.18-83.11
Acrosomal enzyme activity (IU/10^6^ sperm)	978	48.46 (15.69)	15.13-127.65
Seminal plasma total α-glucotase (U/ml)	959	416.93 (191.90)	48.66-1019.84
Seminal plasma GGT (U/ml)	959	2436.08 (836.43)	17.62-4477.56
Seminal plasma fructose (mmol/L)	959	14.24 (7.18)	3.48-53.83
Seminal plasma zinc (mmol/L)	959	2.62 (1.16)	0.14-7.62
Serum TG (mmol/L)	974	1.65 (1.48)	0.05-23.34
Serum TC (mmol/L)	974	4.39 (0.93)	0.54-12.19
Serum LDL (mmol/L)	974	2.59 (0.73)	0.02-5.77
Serum HDL (mmol/L)	974	1.22 (0.26)	0.67-3.04
Serum LH (IU/L)	954	4.25 (2.13)	0.98-25.45
Serum FSH (IU/L)	954	4.84 (2.55)	1.14-26.43
Serum testosterone (nmol/L)	954	13.14 (4.06)	1.80-30.41
Serum estradiol (pmol/L)	954	108.74 (48.63)	3.0-331.0
Serum SHBG (nmol/L)	954	26.02 (10.92)	5.7-70.7
Seminal plasma TG (mmol/L)	887	0.14 (0.11)	0.01-0.90
Seminal plasma TC (mmol/L)	887	0.85 (0.51)	0.01-3.11
Seminal plasma LDL (mmol/L)	887	0.66 (0.34)	0.01-2.70
Seminal plasma HDL (mmol/L)	887	0.32 (0.21)	0.01-1.64
Seminal plasma FSH (IU/L)	396	0.29 (0.26)	0.10-2.13
Seminal plasma testosterone (nmol/L)	396	4.44 (2.15)	1.18-14.57
Seminal plasma estradiol (pmol/L)	396	276.17 (90.61)	62.06-505.73

*TNPMS* Total normal-progressively motile spermatozoa count, *SHBG* sex hormone-binding globulin

Next, we analyzed the correlations between sperm DFI and age, obesity-associated markers, semen parameters, seminal plasma biochemical markers, serum lipids and reproductive hormones, and seminal plasma lipids and reproductive hormones in subfertile men. All these variables were consistent with skewed distribution, so the correlations between these variables were analyzed by Spearman’s rho test, and the obtained results were shown in Table 2. The results showed that Sperm DFI was positively related to age and abstinence duration (*P*<0.001). Sperm DFI was also positively related to semen volume and percentage of abnormal sperm head (*P*<0.001), while negatively related to sperm concentration, progressive motility, sperm motility, percentage of normal sperm morphology, TNPMS, percentage of intact acrosome sperm and acrosomal enzyme activity (*P*<0.001). Sperm DFI was positively related to seminal plasma zinc but unrelated to seminal plasma total α-glucotase, GGT and fructose levels. There was no any significant correlation between sperm DFI and obesity-associated markers such as BMI, WC, WHR and WHtR, and serum lipids levels. However, sperm DFI was positively related to seminal plasma TG and TC levels. Sperm DFI was also positively related to serum LH and FSH levels and seminal plasma FSH and estradiol levels, but unrelated to serum and seminal plasma testosterone levels.

**Table 2:** Non-parametric (Spearman) correlation coefficients (*r*) for relationships between sperm DFI and other parameters

Variables	Number	DFI	
		*r*	*P* value
Age	1010	0.115	<0.001
BMI	1010	-0.051	0.108
WC	1010	-0.065	0.050
WHR	1010	-0.009	0.785
WHtR	1010	-0.057	0.068
Abstinence time	1010	0.171	<0.001
Semen volume	978	0.145	<0.001
Sperm concentration	978	-0.115	<0.001
Total sperm count	978	-0.054	0.091
Progressive motility	978	-0.474	<0.001
Sperm motility	978	-0.487	<0.001
Normal sperm morphology	978	-0.222	<0.001
TNPMS	978	-0.288	<0.001
Abnormal head	978	0.184	<0.001
Intact acrosome	978	-0.191	<0.001
Acrosomal enzyme activity	978	-0.307	<0.001
Seminal plasma total α-glucotase	959	0.089	0.057
Seminal plasma GGT	959	0.080	0.063
Seminal plasma fructose	959	0.002	0.957
Seminal plasma zinc	959	0.137	<0.001
Serum TG	974	0.026	0.411
Serum TC	974	-0.007	-0.820
Serum LDL	974	-0.005	-0.865
Serum HDL	974	0.008	0.814
Serum LH	954	0.134	<0.001
Serum FSH	954	0.113	<0.001
Serum estradiol	954	-0.039	0.234
Serum testosterone	954	-0.042	0.194
Serum SHBG	954	0.038	0.239
Seminal plasma TG	887	0.136	<0.001
Seminal plasma TC	887	0.131	<0.001
Seminal plasma LDL	887	0.013	0.695
Seminal plasma HDL	887	-0.064	0.058
Seminal plasma FSH	396	0.184	<0.001
Seminal plasma estradiol	396	0.099	0.048^*^
Seminal plasma testosterone	396	-0.039	0.433

*TNPMS* Total normal-progressively motile spermatozoa count, *SHBG* sex hormone-binding globulin. *: *P*<0.05. Sperm DFI was positively related to age and abstinence time. Sperm DFI was also positively related to semen volume and the percentage of abnormal sperm head, while negatively related to sperm concentration, progressive motility, sperm motility, the percentage of normal sperm morphology, TNPMS, the percentage of intact acrosome sperm and acrosomal enzyme activity. Sperm DFI was positively related to seminal plasma zinc but unrelated to seminal plasma α-glucotase, GGT and fructose levels. There was no any significant correlation between sperm DFI and obesity-associated markers such as BMI, WC, WHR and WHtR, and serum lipids levels. However, sperm DFI was positively related to seminal plasma TG and TC levels. Sperm DFI was also positively related to serum LH and FSH levels and seminal plasma FSH and estradiol levels, but unrelated to serum and seminal plasma testosterone levels

Although Spearman correlation analysis found that many variables were correlated with sperm DFI, some of them may be confounders. Therefore, we further performed the multivariate regression analysis to assess the variables which were significantly correlated with sperm DFI in Spearman’s rho test. It was found that age, semen volume, sperm concentration, progressive motility, TNPMS and intact acrosome were independently correlated with sperm DFI (Table 3).

**Table 3:** The multivariate regression analysis of the variables correlated with sperm DFI in Spearman correlation (*n*=298)

Variables	Odds Ratio	95% CI	*P* value
Age	0.865	0.785-0.954	0.004
Abstinence time	0.926	0.719-1.193	0.551
Semen volume	0.548	0.390-0.771	0.001
Sperm concentration	0.978	0.962-0.994	0.008
Progressive motility	1.173	1.041-1.323	0.009
Sperm motility	0.985	0.907-1.069	0.713
Normal sperm morphology	0.716	0.487-1.051	0.088
TNPMS	1.307	1.012-1.687	0.040
Abnormal head	0.996	0.947-1.048	0.876
Intact acrosome	1.082	1.006-1.163	0.033
Acrosomal enzyme activity	0.996	0.975-1.019	0.750
Seminal plasma zinc	1.229	0.705-2.142	0.466
Serum LH	0.976	0.768-1.239	0.839
Serum FSH	1.086	0.888-1.328	0.422
Seminal plasma TG	1.677	0.046-60.819	0.778
Seminal plasma TC	0.807	0.224-2.913	0.744
Seminal plasma FSH	0.455	0.075-2.752	0.391
Seminal plasma estradiol	0.987	0.970-1.003	0.111

*TNPMS* Total normal-progressively motile spermatozoa count. Age, semen volume, sperm concentration, progressive motility, TNPMS and intact acrosome were independently correlated with sperm DFI

In addition, for further verifying the correlations of sperm DFI with obesity-associated markers, we set different groups according to the criteria of obesity, and compared sperm DFI based on the dichotomized analyses for BMI, WC, WHR and WHtR (Table 4). We found that there was no any significant difference in sperm DFI among different groups, demonstrating again that sperm DFI was not correlated with obesity-assocaited markers.

**Table 4:** Comparison of sperm DFI based on the dichotomized analyses for BMI, WHR, WC and WHtR

Variables		Number	DFI (%)
BMI (kg/m^2^)	<18.5	20	17.60 ± 10.61
	18.5-23.99	507	18.91 ± 12.43
	24-27.99	389	18.63 ± 12.77
	≥28	94	17.17 ± 11.76
	*F*		0.556
	*P*		0.644
WC (cm)	<90	807	18.76 ± 12.50
	≥90	203	18.04 ± 12.33
	*t*		0.548
	*P*		0.459
WHR	<0.9	747	18.75 ± 12.54
	≥0.9	263	18.24 ± 12.26
	*t*		0.321
	*P*		0.571
WHtR	<0.5	686	19.00 ± 12.71
	≥0.5	324	17.80 ± 11.91
	*t*		2.037
	*P*		0.154

## Discussion

The determination of sperm DNA damage, as an important supplement to semen routine determination strategies, has been applied in some clinical andrology laboratories worldwide. What factors may lead to sperm DNA damage remains one of the major concerns. There were increasingly accumulated evidence for the correlation between obesity and male subfertility. It was reported that obesity was closely related to male subfertility [16]. Obesity and related abnormal lipids metabolism and the change of reproductive hormones might lead to the decrease of sperm quality [3, 4]. However, it has little reports whether obesity and related abnormal lipids metabolism and reproductive hormones may lead to the increase of sperm DNA damage. Based on these, we prospectively investigated the correlations between sperm DFI and obesity-associated markers, serum and seminal plasma lipids and reproductive hormones, as well as, age, abstinence time and semen parameters.

First, as expected, our results showed that sperm DFI was positively related to age and abstinence time in subfertile men, which was consistent with other reports [17–19]. Moreover, it was reported that sperm DFI in men with age ≤ 35 years was significantly lower than that in men with age between 36-39 years and above 40 years, and no significant difference in the latter two [17, 18], indicating that the proportion of sperm with DNA damage increased with increasing age, and that the best child-bearing age in males would be before 35 years old. In addition, in order to guarantee sperm quality, the abstinence time in males should not too long.

Next, we investigated the correlations between sperm DFI and semen parameters and seminal plasma biochemical markers. Similar to others’ results [20–22], we found that sperm DFI was positively related to semen volume and percent of abnormal head sperm (*P*<0.001), while negatively related to sperm concentration, PR, sperm motility, TNPMS, percent of normal sperm morphology, percent of intact acrosome and acrosomal enzyme activity (*P*<0.001). It was reported that sperm DFI was independent of and could predict male fertility better than routine semen parameters [23]. Our data showed that sperm DFI was almost significantly related to all parameters reflecting semen quality, especially TNPMS, indicating that sperm DNA damage might be key factor leading to the decrease of semen quality, that is, abnormal semen parameters.

We found that sperm DFI was positively related to seminal plasma zinc level (*P*<0.001), but unrelated to seminal plasma total α-glucosidase, GGT and fructose levels. Seminal plasma total α-glucosidase, GGT and fructose levels may be used to evaluate the secretory function of epididymis, prostate and seminal vesicle, indicating that the auxiliary gonad might be less effect on sperm DNA damage, and that sperm DNA damage might be existed before sperm transferred into epididymis. It was reported that zinc played an important role not only in normal testis development but also in spermatogenesis and the maintenance of sperm motility [24, 25]. However, the excess of zinc in seminal plasma may accumulate in sperm, produce adverse effect on sperm DNA quality [26], and reduce sperm motility and survival [25]. It is indicated that the excess of seminal plasma zinc might lead to sperm DNA damage, and that the supplement of zinc in clinic should be appropriate.

Further investigations on the relationship between obesity-associated markers and sperm DFI showed that there was no any correlation between sperm DFI and BMI, WC, WHR and WHtR. Moreover, there was no any correlation between sperm DFI and serum lipids levels. This was consistent with our early investigation results, that is, obesity-associated markers could not predict semen quality in subfertile men [3], and there was no any correlation between serum lipids levels and semen parameters [4]. Furthermore, recently, Bandel *et al*. [5] found that there was no any correlation between BMI and sperm DNA integrity, which was similar to our results. However, we found that sperm DFI was positively related to seminal plasma TG and TC levels. As observed in our early study [4], unlike lipids levels in serum, TG, TC, LDL and HDL levels in seminal plasma were all negatively related to some of semen parameters. Likewise, this study demonstrated that the increase of seminal plasma TG and TC levels might lead to increased sperm DFI, indicating that the elevated lipids levels in seminal plasma might have adverse effect on sperm quality. However, the detail mechanism needs to be further elucidated.

As obesity could lead to the change of reproductive hormones, we also investigated the correlations between sperm DFI and seminal plasma reproductive hormones levels. The results showed that sperm DFI was positively related to not only serum LH and FSH levels but also seminal plasma FSH and E2 levels (*P*<0.001), but unrelated to serum and seminal plasma testosterone. Our early investigations had demonstrated that serum LH and FSH levels were negatively correlated with sperm concentration and percent of normal sperm morphology [3]. Moreover, it was reported that the change of serum LH and FSH levels had adverse effect on male fertility [27], and that serum FSH level was the risk factor of sperm DFI [28], which demonstrated that serum FSH and LH levels might have adverse effect on sperm maturation, and might interfere sperm DNA integrity. In addition, we also detected the reproductive hormones levels in seminal plasma, and found that sperm DFI was also positively related to seminal plasma FSH and E2 levels, indicating that the higher FSH and E2 levels in local genital tract likewise had adverse effect on sperm DNA integrity. All these demonstrated that reproductive hormones levels might influence on the integrity of sperm chromatin and then on sperm fertilizing ability [29].

Although Spearman correlation analysis found that many variables were correlated with sperm DFI, the multivariate regression analysis for the variables which were significantly correlated with sperm DFI in Spearman’s rho test showed that only age, semen volume, sperm concentration, progressive motility, TNPMS and intact acrosome were independently correlated with sperm DFI. Due to the less semen volume for each sample, not all the parameters of each sample investigated in the study were detected. A total of 298 samples were analyzed all the parameters. Perhaps it was the lower size of samples that led to the difference in the results between Spearman correlation analysis and multivariate regression analysis. Therefore, whether sperm DFI is correlated with abstinence time, sperm motility, normal sperm morphology, abnormal head, acrosomal enzyme activity, serum LH and FSH, and seminal plasma zinc, TG, TC, FSH and estradiol should be further demonstrated by a large size of samples.

In the future, the mechanisms that independent correlation factors lead to sperm DNA damage should be clarified, while the potential correlation factors should be further demonstrated. The key goal to investigate sperm DFI-related factors is to prevent sperm DNA damage during spermatogenesis and sperm maturation. Therefore, the intervention experiments to some correlation factors may verify their effects on sperm DNA damage.

## Conclusions

In conclusion, there are many potential factors associated with sperm DFI, including age, abstinence time, spermatogenesis and maturation, seminal plasma lipids and reproductive hormones levels. However, the potential effects of seminal plasma lipids and reproductive hormones on sperm DNA damage need still to be demonstrated by the studies with scientific design and a large size of samples.

## Additional file

Additional file 1. Raw data for DFI and its related variables. (XLS 388 kb)

## Abbreviations

DFI: sperm DNA fragmentation index
SCSA: sperm chromatin structure assay
AO: acridine orange
TSC: total sperm count
PR: progressive motility
TNPMS: total normal-progressively motile sperm count
NSM: percent of normal sperm morphology
CASA: computer-aided sperm analysis
BMI: body mass index
WHR: waist-to-hip ratio
WC: waist circumference
WHtR: waist-to-height ratio
TG: triglyceride
TC: total cholesterol
LDL-C: low density lipoprotein cholesterol
HDL-C: high density lipoprotein cholesterol
GGT: γ-glutamyl transpeptidase
SHBG: sex hormone binding globulin
FSH: follicle stimulating hormone
E2: estradiol
T: testosterone
TT: total testosterone
c-PAHs: polycyclic aromatic hydrocarbons
ZEA: zearalenone
